# Cyanoacrylate Glue Masquerading as an Obstructive Calculus: Rare Sequelae of Angioembolization for Renal Pseudoaneurysm

**DOI:** 10.7759/cureus.35135

**Published:** 2023-02-18

**Authors:** Siddharth K Prasad, Omang Agrawal, Ankur Mittal, Vikas K Panwar, Deepak Dubey

**Affiliations:** 1 Urology, All India Institute of Medical Sciences Rishikesh, Rishikesh, IND

**Keywords:** cyanoacrylate glue complication, calculus secondary to glue migration, glue emobolisation complication, arteriocalyceal fistula, glue migration post angioemobilzation

## Abstract

Renal pseudoaneurysm is a well-known albeit rare vascular complication following renal trauma, percutaneous interventions, renal biopsy, and partial nephrectomy. Angioembolization has become an effective treatment option for pseudoaneurysm using Cyanoacrylate glue, Gel-foams, Micro-coils, polyvinyl alcohol, etc. We herein present a 20-year-old gentleman with infected left hydroureteronephrosis secondary to an impacted foreign body in a ureter, specifically, down-migrated cyanoacrylate glue. This is two weeks following glue angioembolization for a left upper polar segmental renal artery pseudoaneurysm secondary to stab injury. He underwent a successful left-side ureteroscopic extraction of this polymerized glue, following which his symptoms subsided. These complications of glue migration following angioembolization are infrequent, and reports of it are scarce in the literature. Stringent follow-up and timely intervention are essential to mitigate disastrous outcomes.

## Introduction

Renal pseudoaneurysm is a vascular lesion that arises when an injury results in damage to one or more arterial wall layers, namely the intima, media, and adventitia. This results in a contained hematoma surrounded by connective tissue and reactive fibrosis, forming an aneurysmal sac [1]. They may be completely asymptomatic or present as flank pain, gross intermittent hematuria, hypertension, renal function deterioration, etc. Angioembolization has become an effective treatment option as it increases the chance of renal tissue preservation and minimizes that of nephrectomy. Cyanoacrylate glue, Gel-foams, Micro-coils, and Polyvinyl alcohol have been used for the same and have demonstrated equivalent efficacy with the success of over 90%. We herein report a rare complication of glue angioembolization done for renal pseudoaneurysm.

## Case presentation

**Figure 1:**
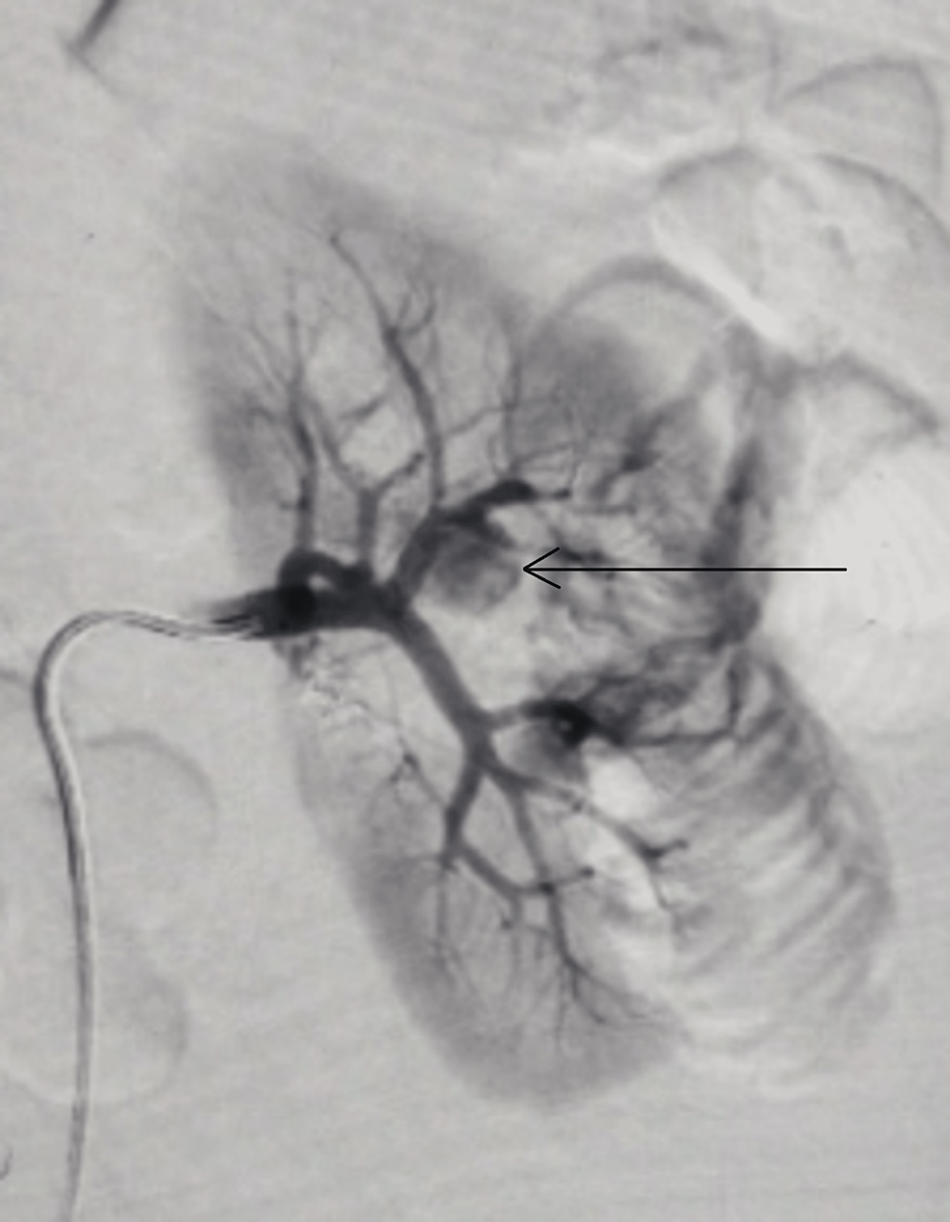
Renal angiography suggestive of renal artery pseudoaneurysm (shown by arrow)

**Figure 2:**
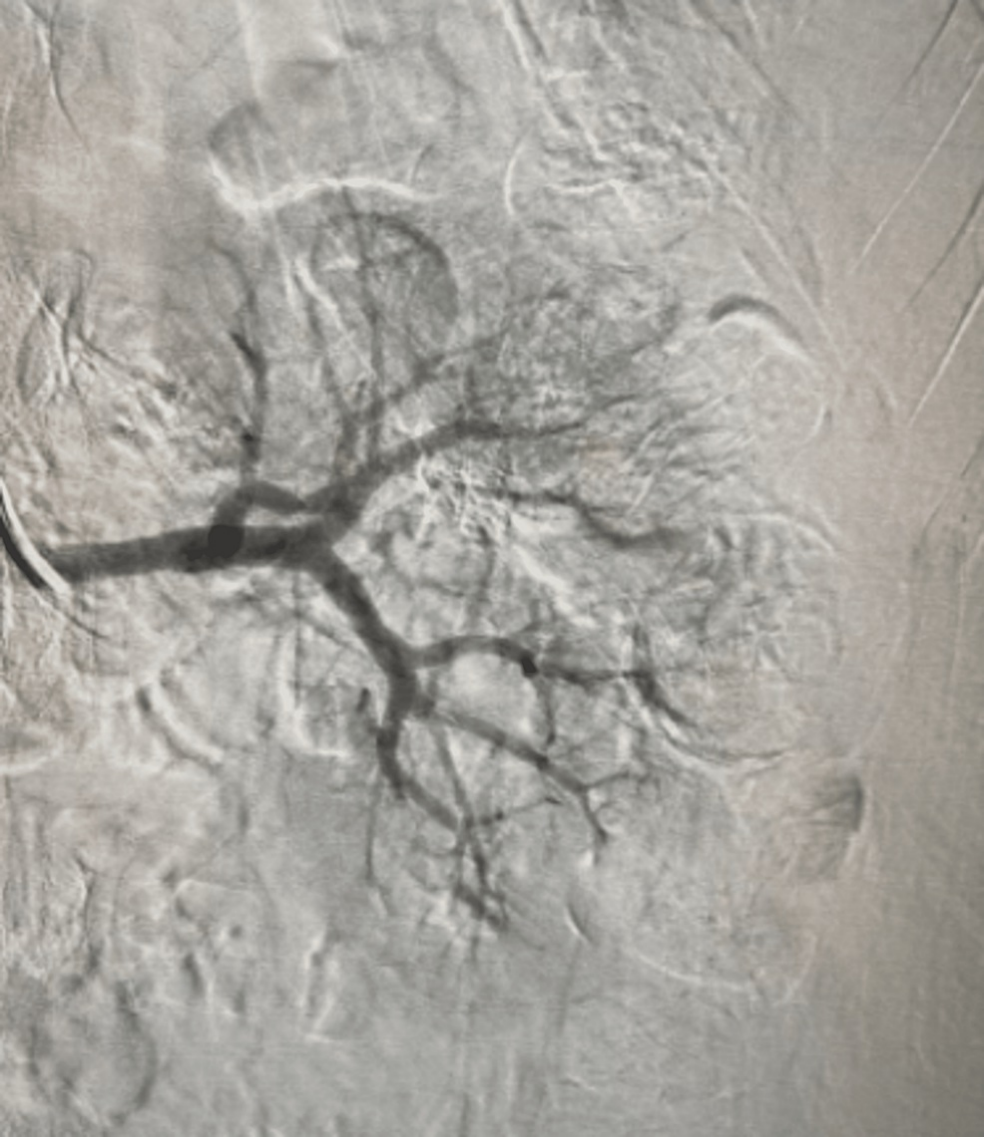
Renal angiography post-glue embolization of pseudoaneurysm

**Figure 3:**
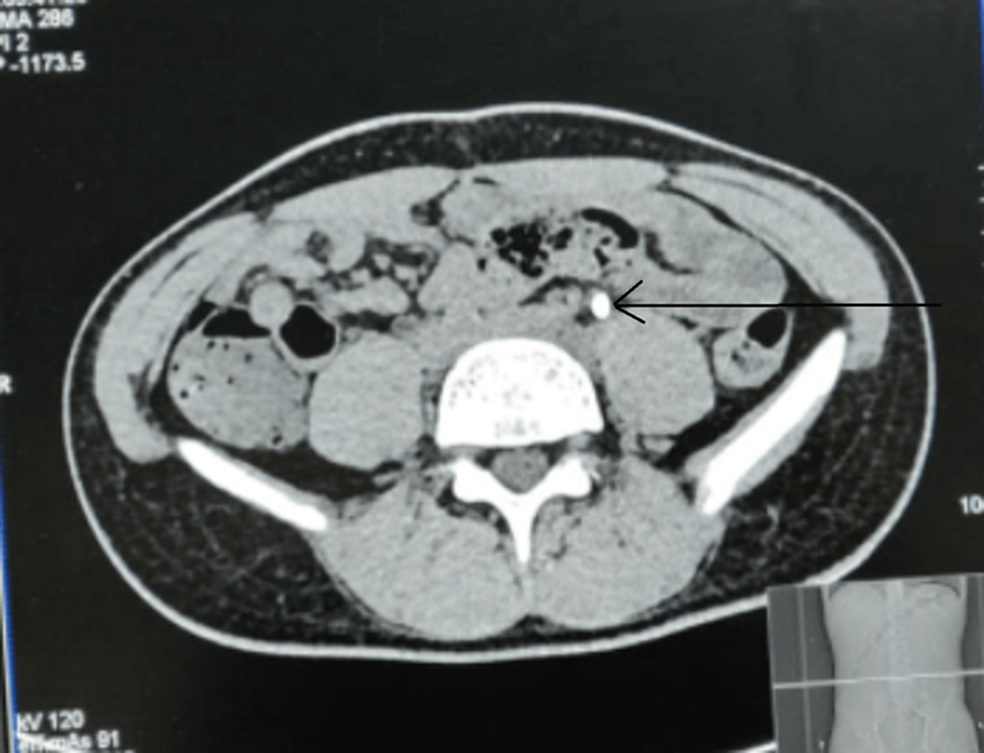
NCCT KUB film showing down migrated glue particles in the left ureter masquerading as left ureteric calculus (shown by black arrow) NCCT KUB: Non-contrast computerized tomography scan of the kidneys, ureter, and bladder

A 20-year-old man presented with episodes of intermittent hematuria and dull aching left flank pain for one month. He had a history of stab injury to the left flank. On evaluation, CT urography with CT renal angiogram revealed a pseudoaneurysm of approximately 1.5cm x 1cm in the left upper polar segmental renal artery. There was normal contrast uptake in the left renal parenchyma and prompt excretion of contrast with around 100 ml of clots in the urinary bladder clots. Once stabilized, he underwent left renal angiography and glue angioembolization of the pseudoaneurysm with clot evacuation (Figures 1, 2). His hematuria subsided, and he was discharged. After two weeks of an asymptomatic period, he presented to the emergency with fever, left flank pain, and renal angle tenderness. USG KUB was suggestive of left-sided infected hydronephrosis. X-ray KUB, to our surprise, was indicative of a radiodense shadow in the region of the upper ureter. A non-contrast computerized tomography scan of the kidneys, ureter, and bladder was done, confirming the findings of the X-ray and showing a hyperdensity of size around 1 cm in the left upper ureter with proximal hydroureteronephrosis (Figure 3). Given infected hydronephrosis and features of frank sepsis, he underwent percutaneous nephrostomy placement that drained turbid urine and was started on adequate intravenous antibiotics per culture sensitivity.

**Figure 4:**
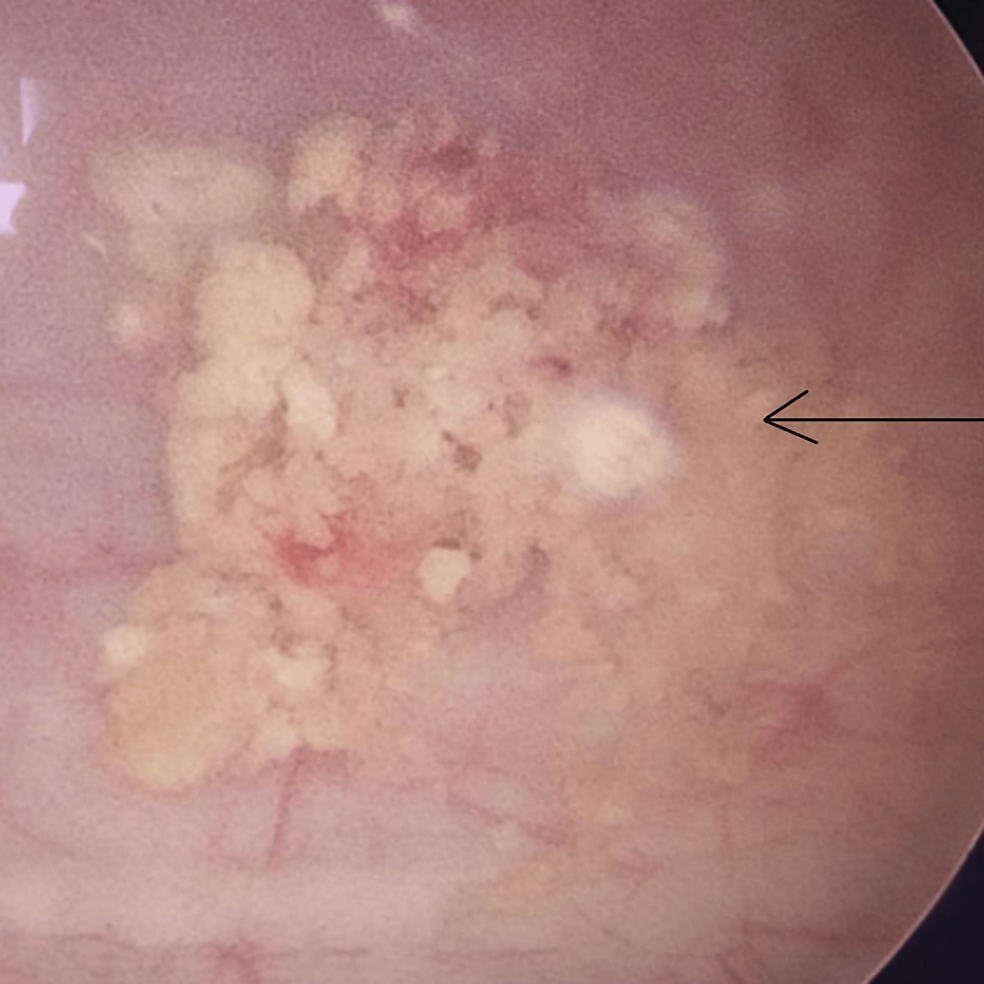
Cystoscopy image of cyanoacrylate glue particles in bladder post ureteroscopic lithotripsy (shown by arrow)

Later, he underwent left-sided Ureteroscopic lithotripsy, and polymerized Cyanoacrylate glue was found in the upper ureter (Figure 4). The postoperative stay was uneventful. The left DJ stent was removed after four weeks, and presently patient is doing well on follow-up.

## Discussion

Minimal invasive interventions have become a go-to strategy for treating renal artery pseudoaneurysms, no matter what the etiopathogenesis. With the improvement in technology, the coaxial catheter and the micro-catheter system play a crucial role in super-selective catheterization of small caliber branches of the renal artery. This helps in minimizing the infarction and leads to maximal renal tissue preservation [2]. Various agents have been used for embolization which can be sub-specified as Absorbable and non-absorbable. Absorbable agents include Gelfoam (Pharmacia & Upjohn Company, Pfizer, NY), Curaspon (Cura Medical BV, Amsterdam, The Netherlands), and Gelitaspon (Gelita Medical BV, Amsterdam, The Netherlands). These get absorbed within three weeks to three months of instillation and have a deformable shape that assists in instillation through micro-catheters [3]. The non-absorbable agents include inert particles with different sizes ranging from 100 to 1,000 μ like polyvinyl alcohol (Avalon, Unipoint Lab., High Point, NC), acrylic polymer microspheres impregnated with gelatin (Embosphere Microspheres, Biosphere Medical, Rockland, MA), and polyvinyl alcohol microspheres (Beadblock, Terumo, Leuven, Belgium). There are Liquid non-absorbable agents like N-butyl 2-cyanoacrylate-type biological glue (Histoacryl, B/Braun, Tuttlingen, Germany, or Glubran 2, GEM SRL, Viareggio, Italy), alcohol solutions of polypeptides associated with a radiopaque agent (Ethibloc, Ethnor Laboratories, Ethicon, Norderstedt, Germany) and high concentrations of ethanol (between 95 and 99%) [4]. Metallic coils, occluders like Amplatzer Vascular Plug (AGA Medical Corporation, Plymouth, MN), and detachable balloons have also been tried for proximal vessel occlusion.

The cyanoacrylate glue has the property to polymerize when it comes in contact with an ionic medium, especially blood, making it one of the most popular agents to be used. The polymerization timing depends on the dissolution with an oily contrast medium such as Lipiodol (Therapex, E-Z-EM, Montreal, Canada). Various adverse events accompany the embolization procedure, like pain, arterial hypertension, post-embolization syndrome, and kidney failure. There are chances of accidental embolization to distal vasculature, pulmonary vessels, and pelvicalyceal system (if there is an arterio- calyceal communication). Owing to the small size and varied nature of these embolization agents, in our case as well, the cyanoacrylate glue made its way through the arterio-calyceal fistula into the pelvicalyceal system and migrated down into the proximal ureter. After polymerization, it got impacted and lead to proximal hydroureteronephrosis. Chen WJ et al. reported a case of glue migration to the ureter after trans-arterial embolization of renal pseudoaneurysm, which passed out spontaneously and did not require any intervention [5]. Kumar S et al. [6] also reported a case in which a patient presented with ureteric stones formed around a coil that was used for angioembolization of a pseudo-aneurysm formed following percutaneous nephrolithotomy done nine years back, getting our attention to the fact that the coil eroded its way into the pelvicalyceal system and proved to be a nidus for stone formation. Keeping all these things in mind, it is highly pertinent to stringently follow up with the patient after trans-arterial embolization with any of these agents as described previously. They may present with a rare scenario that might hamper renal function.

## Conclusions

While angioembolization is a first-line treatment option for renal artery pseudoaneurysm, each agent utilized has a diverse complication, albeit rare. There are chances of an arterio-calyceal fistula in penetrating renal trauma, which might allow glue migration from the artery to the pelvicalyceal system and ureter. The polymerized glue may act as an obstructing lesion mimicking a calculus as in our case. Once diagnosed, the management is safe and efficacious by the endourological intervention.
